# Admission criteria and academic performance in medical school

**DOI:** 10.1186/s12909-023-04251-y

**Published:** 2023-04-21

**Authors:** Ahmad Tamimi, Mariam Hassuneh, Iskandar Tamimi, Malik Juweid, Dana Shibli, Batool AlMasri, Faleh Tamimi

**Affiliations:** 1 Section of Neurosurgery, University of Jordan, Amman, Jordan; 2 College of Dental Medicine, QU Health, Qatar University, Doha, Qatar; 3 Department of Radiology and Nuclear Medicine, University of Jordan, Amman, Jordan; 4 Department of Orthopedic Surgery, Universidad de Malaga, Malaga, Spain

**Keywords:** Medical education, Admission system, Academic performance, High School performance, Predictive value, Jordan

## Abstract

**Background:**

Different variables have been used to predict the academic performance of students in medical schools. The aim of this study was to assess the effect of demographics, admission system, and high-school background on the academic performance of medical students.

**Methods:**

We conducted this longitudinal cohort study on 808 students admitted to the Faculty of. Medicine at the University of Jordan (Amman, Jordan), in the years 2012 and 2013. Admission pathway, and academic performance data were collected and analyzed.

**Results:**

A total of 808 students [i.e., 426 (52.7%) females, and 382(47.3%) males] were identified. Admitted students were holding 17 different types of high school degrees, and were accepted through 6 different quota pathways (open competition [National unified admission], underprivileged [“Makrumah”], parallel, children of university staff, international students, and others). Students admitted through the open competition and the underprivileged quota(Makrumah) were more likely to graduate on time and had higher graduation grades while students admitted through the parallel, international and others quota were more likely to fail and had lower graduation grades. Regarding highs school degrees, the students that were more likely to graduate were those with IB and the Jordanian high school degrees. The highest graduation GPA was for IB students followed by SAT, IGCSE as well as Jordanian and Syrian high school degrees respectively. IB, Jordanian, Kuwaiti and IGSC high school grades were significantly correlated with the graduation GPA.

**Conclusions:**

Admission criteria such as type of high school degree and grades as well as admission pathways can predict the likelihood to graduate and the graduation GPA of medical students. Open competition and underprivileged admission pathways as well as IB, IGCSE and Jordanian high school degrees seem to be better predictors of student performance in the medical school.

**Supplementary Information:**

The online version contains supplementary material available at 10.1186/s12909-023-04251-y.

## Introduction

Medicine is one of the most demanded careers worldwide, and access to medical schools is highly competitive. Consequently, medical schools face significant challenges in objectively selecting the best applicants [1, 2], and the admission process they use can have an important impact on the academic performance of students in medical schools [3, 4]. Thus, to recruit the best students medical schools use a variety of cognitive and non-cognitive criteria which may include previous academic performance, aptitude tests, interviews, and personal statements [5, 6].

Non-cognitive selection criteria such as aptitude tests and interviews are controversial [2, 5–12], however, high school performance seems to be a strong predictor for academic performance in medical schools [13]. Thus, many medical schools rely heavily on student high school grades in their admission process, specially when students are admitted directly from high school. However, there is a wide variety of high school degrees available around the world, and it is not clear which ones are better predictors for student performance in medical schools.

Demographic and socioeconomic factors have also been studied as possible predictors of student performance in medical schools [7–10]. Although underprivileged students face more challenges in getting accepted, once in the program, student socio-economic status seems to have little or no correlation with their performance [14, 15]. On the other hand, age and ethnicity, have been found to predict student performance in medical schools [13, 16]. To address these variations, many medical schools have set-up admission pathways that involve quota designed to account for students’ socioeconomic and demographic conditions.

The Faculty of Medicine of Jordan University(Amman, Jordan) is one of the most prestigious in the middle east, and the quality of its program is attested by the excellent performance of its graduates in the United States Medical Licensing Examination (USMLE), with 90.96% of students passing step 1, and 89.18% passing step 2 on their first attempt [17, 18]. This faculty, as well as many other faculties in the middles east, receives application of student from many different countries, holding a wide range of high school degrees (i.e. Saudi high school certificates, Kuwaiti highs school certificates, etc.) and international certificates (ACT, IB, etc.). In addition, at the University of Jordan, students can apply through different admission pathways that involve various quota, including a quota for underprivileged students, as well as another one for international students. This poses a challenge to the admission process on how to factor the variability, but it also creates a unique opportunity for comparisons between the different high school programs and admission pathways.

Within the limit of our knowledge, there is a gap in the literature on the effect of different high school certificates and admission pathways on the performance of medical students in university.

Thus, this study was designed to address the hypothesis that admission pathway and type of high school degree considered for university admission in a Jordanian medical school could predict student academic performance in the medical school. This was done by investigating the performance of medical students admitted at Jordan University Faculty of Medicine.

## Materials and method

This study protocol was assessed and approved by the Institutional Review Board (IRB) at Jordan University Hospital (#3796/2020/67) and Ethical Committee at the Jordan University Hospital and Faculty of Medicine at Jordan University (#10/2020/20873), and it was granted an exemption from the need for informed consent in accordance with the Declaration of Helsinki and the ethical guidelines and regulations of Jordan University Hospital, the University of Jordan, and the national laws that define consent regulations. To ensure privacy, the data linkage and appropriate anonymization were carried out by a data protection official. All analyses were part of the quality management of Jordan University.

### Study population

This was a retrospective cohort longitudinal study performed on medical students who were admitted to the Faculty of Medicine of the University of Jordan (Amman, Jordan) during two consecutive academic years (2012 and 2013). We excluded students admitted before or after these dates.

### Admission pathways

The admission system in our medical schools includes the following quota:

i) The Open National Unified Admission (NUA) track. This is the most competitive pathway, and it consists of a the national competence exam organized by the Ministry of Education of Jordan.
ii) The quota for underprivileged students (a.k.a. Makrumah pathway [M]. This is a “quota” that mainly includes among others the children of servicemen in the military, as well as children of government employees in the Ministry of Education.
iii) Children of university employees (UEC).
iv) The parallel track(P). This is a quota for students with lower scores that are required to pay higher registration university fees.
v) The international pathway (I). This is a quota for international students that are also required to pay higher university fees
vi) Others(O). This quota includes students admitted through international cooperation agreements, and students from low-income families coming from remote areas of the country.

In this study, Jordanian students and non-Jordanian students from 14 different countries were included. Students were admitted based solely on the grades of their high school degree. The high school degrees considered were the Jordanian General Secondary Certificate (JGSC), the International General Certificate of Secondary Education (IGCSE), the Scholastic Assessment Test (SAT), the International Baccalaureate (IB), as well as high school degrees from Bahrein, Canada, Egypt, Emirate, Iraq, Israel, Kuwait, Libya, Malaysia, Qatar, Palestine, Saudi Arabia, Syria, and Yemen. The following demographic and admission parameters were analyzed and correlated with the odds of graduating and the graduation GPA: age, gender, type of high school certificate, high school performance, admission system (i.e., national unified or others).

The JGSC is a standard national examination administrated by the Ministry of Education of Jordan that is performed at the end of secondary education. The students who meet the minimum requirements in the science track of JGSC (i.e., a score of 85%) or equivalent are eligible to apply for medical schools in Jordan. This is the only academic criteria in the Jordanian admission system; there is no additional cognitive or non-cognitive tests.

### Student graduation

Students were considered to have graduated on time and received their medical degree (MD) only upon completing all the requirements of the faculty´s curriculum within 6 years from the time of admission [17]. The program is divided into two periods: 3-years of basic medical sciences and 3-years of clinical sciences. The first period includes general basic sciences (e.g., general biology, general chemistry), basic medical sciences (e.g., anatomy, pharmacology) and elective university courses (lectured in non-medical departments). During this period, students are assessed at the end of each course by a series of multiple-choice exams and skill labs. The second period includes rotations at different clinical departments. Student evaluation at this stage relays on theoretical multiple-choice exams, patient management problems, oral exams, and objective structured clinical examinations. At the end of the 6^th^ year, students are assessed by a series of comprehensive theoretical and practical exams. The clinical assessment of the students is conducted by committees composed of teaching staff, and national and external examiners (i.e., Jordanian medical schools, Arab and Western universities). Research projects required for graduation are evaluated by a faculty committee during the last semester of the program.

The academic performance during the different stages of the program was determined using the graduate point average (GPA), which ranges from 0.0 to 4.0 points [i.e., excellent (3.65–4.00); very good (3.00–3.64); good (2.50–2.99); pass (2.00–2.49); weak (1.00–2.00); fail < 1.00]. Students may only pass with a minimum accumulative score of 2.00 per year.

### Statistical analysis

The statistical analyses were performed using the statistical analysis software IBM, SPSS Statistics 20.0 (IBM Inc., Chicago, IL, USA). Continuous variables were assessed for normal distribution using the Shapiro–Wilk test. For normally distributed data, differences between groups were analyzed using ANOVA test with Bonferroni correction while for data not distributed normally statistical differences were assessed with Kruskal Wallis test for comparison of multiple groups and Mann–Whitney test for comparison of 2 groups. Dichotomous variables were analyzed using the Chi-square test and logistic regression adjusted for potential confounders. Differences were considered significant if the two-tailed *p*-value was less than 0.05.

## Results

A total of 808 students were included in this study; 303 were admitted in 2012, and 505 in 2013. There were slightly more female students than male students admitted [i.e., 426 (52.7%) females, and 382 (47.3%) males]. A total of 626 (77.5%) students eventually graduated from the Faculty of Medicine (Table 1). Logistic regression analysis revealed that students admitted through the parallel pathway and International students were significantly less likely to graduate on time, while students admitted through the national unified admission and through the quota for university employees were more likely to graduate on time. The mean graduation age was 25.9 ± 0.8 years, and more female students graduated than male students [i.e., 356(56.9%) females, and 270(43.1%) males] (Table 2).

**Table 1:** Association between student admission pathway and student graduation

Admission pathway	Applications	Admission			Graduation			
	N	N	per application	per pathway	N students ^a^ (%)	Odds of graduating on time		
						OR(95%CI)	AOR(95%CI) *	P*
NUA	7468	113	1.5%	14%	112(99. 1%)	39.44(5.47–284.50)	**10.00(1.26–79.25)**	**0.029**
P	1987	143	7.3%	17.7%	133(93. 0%)	0.22(0.11–0.42)	**0.43(0.20–0.93)**	**0.03**
I	NA	234	NA%	29.0%	141(60. 3%)	0.28(0.20–0.39)	**0.38(0.23–0.65)**	** < 0.001**
M	3428	112	3.2%	13.9%	111(99. 1%)	39.01(5.41–281.44)	4.64(0.61–35.34)	0.140
UEC	NA	67	NA	8.3%	66(98.5%)	21.33(2.94–154.79)	**7.29(1.06–64.70)**	**0.044**
Others	NA	139	NA	17.2%	63(45.3%)	0.16(0.11–0.23)	0.62(0.34–1.14)	0.123
Total		808	NA	100.0%	626(77. 5%)			

Abbreviations: *NUA* National Unified, *P* Parallel, *I* International, *M* Makrumah, *UEC* University Employees Children

^a^ Students Graduating on time; *OR* Odd ratio, *AOR* Adjusted odd ratio

^*^Logistic regression adjusted to high school score and high school type, *NA* Data not available

**Table 2:** Gender distribution of admitted students as a function of admission system

Admission pathway	Female	Male (%)	OR(95%CI)	*p*	AOR(95%CI)^*^	P*
National Unified system (NUA)	76(67.3%)	37(37.7%)	1	0.001	1	
Parallel system(P)	74(51.7%)	69(48.3%)	1.92(1.15–3.20)	**0.009**	**1.85(1.07–3.20)**	**0.027**
International system (I)	88(37.6%)	146(62.4%)	3.41(2.12–5.47)	** < 0.001**	**2.91(1.64–5.16)**	** < 0.001**
Makrumah system (M)	77(68.8%)	35(31.3%)	0.93(0.53–1.64	0.461	0.89(0.50–1.60)	0.704
University Employees Children (UEC)	41(61.2%)	26(38.8%)	1.30(0.69–2.44)	0.423	1.29(0.67–2.49)	0.447
Others	70(50.4%)	69(49.6%)	2.03(1.21–3.39)	**0.005**	**0.91(0.33–2.57)**	**0.864**

Abbreviations: *NUA* National Unified, *P* Parallel, *I* International, *M* Makrumah, *UEC* University Employees Children, *OR* Odd ratio, *AOR* Adjusted odd ratio

^*^Logistic regression adjusted to high school score and high school type

### Admission pathway

**Fig. 1:**
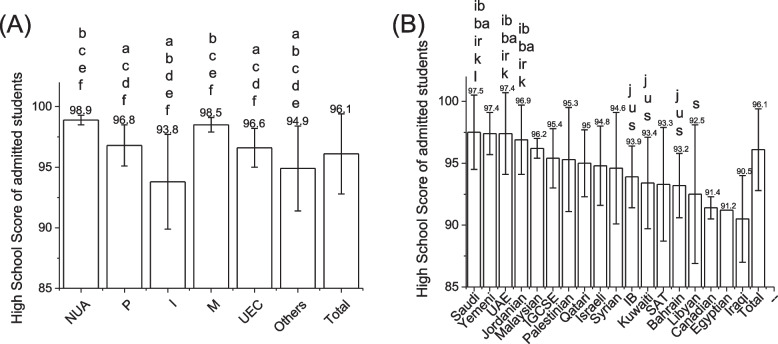
**A** Column bar graph showing the average and standard deviation of high school grades per each admission pathway. Abbreviations: National Unified (NUA), Parallel (P), International (I), Makrumah (M), University Employees Children (UMS). Statistical analysis were done with ANOVA with Bonferroni correction. a: Significantly different from the National Unified admission group, b: significantly different from parallel-group, c: significantly different from International students group, d: significantly different from Makrumah group, e: significantly different from University employees group, f: significantly different from “others” group. **B** Column bar graph showing the average and standard deviation of high school grades per each highs school degree. Statistical differences were assessed with Kruskal Wallis test: s: different than Saudi; K: different than Kuwait; Q different than Qatar, ba: significantly different than Bahrain; u: significantly different than UAE; J significantly different than Jordanian Secondary general exam; L: significantly different than Libya; Irak; IR: significantly different than Irak; I: significantly different than IG; ib: significantly different than IB

Most students were admitted through the international system (*n* = 234[29.0%]) followed by the parallel system (*n* = 143[17.7%]), the “others” category, the open unified admission system(113[14.0%]), the “makrumah” system (*n* = 112[13.9%]),and the children of university employees(67[8.3%]). High school scores were highest for the open unified admission system (98.9 ± 0.4%) and “makrumah” admission system (96.8 ± 1.7%) followed by the university employees children (96.6 ± 1.6%), the “others” category, the international students (94.9 ± 3.5%) and the parallel system(93.8 ± 3.9%) (Fig. 1). More females than males were admitted regardless of the admission system except for students admitted through the international pathway who included more males than females (Table 2). Odd ratio analysis revealed there were significantly more female students accepted through the national unified system than through the international pathway, the parallel pathway and the “other” pathways. These differences remain significant after adjusting for the type of high school diploma in the logistic regression analysis (Table 2).

Students admitted through the parallel, the international and the “others” pathways were less likely to graduate. Failure to graduate was significantly highest for students admitted through the “others” category followed by international students (Table 1). Upon graduation, students admitted through the open National unified admission system and the quota for underprivileged students (Makrama) had significantly higher graduating GPA (3.30 ± 0.04 and 3.25 ± 0.04 respectively) than all other admission pathways. The students with the lowest graduating GPA were those admitted through the international admission system (2.87 ± 0.04) and through the “other “admission system (2.76 ± 0.05). There were no significant differences between male and female students graduating GPA except for students admitted through the Makrumah system (*P* = 0.004) (Table 3).

**Table 3:** Graduating student's GPA according to student admission path

Admission pathway	All students		Female students		Male students		p**
	N(%)	Mean(SE)^*^	n	Mean(SE)	n	Mean(SE)	
National Unified Admission(NUA)	112(18%)	3.30(0.04)^b,c,e,f^	76	3.29(0.05)	36	3.31(0.07)	0.794
Parallel system(P)	133(21%)	3.01(0.04)^a,c,f^	70	3.03(0.05)	63	2.99(0.05)	0.565
International system(I)	141(23%)	2.87(0.04)^a,d^	55	2.86(0.05)	86	2.88(0.05)	0.73
Makrumah system(M)	111(18%)	3.25(0.04)^b,c,e,f^	77	3.18(0.05)	34	3.42(0.06)	**0.004**
University Employees Children (UEC)	66(11%)	2.95(0.04)^a,d^	41	2.99(0.06)	25	2.87(0.05)	0.179
Others(O)	63(10%)	2.76(0.05)^a,b,d^	37	2.76(0.06)	26	2.77(0.08)	0.945
All	626		356		270		

^*^ Statistical comparison between groups were done with ANOVA test with Bonferroni correction: a: significantly different than general admission, b: significantly different than parallel admission, c significantly different than international admission, d significantly different than makrumah, e significantly different than children of University employees, f: significantly different than others

^**^Statistical comparison between female and male students were done using Mann–Whitney test

### High school degrees

Regarding the high school degrees considered for admission, there were 445 (55.1%) students admitted with a Jordanian JGSC certificate and 363 (44.9%) with other certificates. This included.

IGCSE [*n *= 63 (7.8%)], IB [*n *= 16 (2.0%)], SAT [*n *= 12 (1.5%)]; as well as high school degrees from Bahrein [*n *= 12 (1.5%)], Canada [*n *= 3 (0.4%)], Egypt [*n *= 1 (0.1%)], United Arab Emirates [*n *= 13 (1.6%)], Iraq [*n *= 17 (2.1%)], Israel [*n *= 9 (1.1%)], Kuwait [*n *= 54 (6.7%)], Libya [*n *= 9 (1.1%)], Malaysia [*n *= 25 (3.1%)], Palestine [*n *= 9 (1.1%)], Qatar [*n *= 7 (0.9%)], Saudi Arabia [*n *= 111 (13.7%)], Syria [*n *= 12 (1.5%)], and Yemen [*n *= 2 (0.2%)] (Table  4).

**Table 4:** Association between high school degree and student graduation from the medical school

High school degree	N admitted students	N graduated students (%)	Odds of graduating on time	
			OR (95%CI)	P*
Jordanian	445	415(93.3%)	0.10(0.07–0.15)	** < 0.001**
IGCSE	63	55(87.3%)	0.48(0.22–1.02)	0.052
SAT	12	975.0	1.03(0.28 -3.79)	0.962
IB	16	16(100.0%)	NC	**0.029**
Bahrain	12	8(66.7%)	1.74(0.52–5.83)	0.367
Canadian	3	2(66.7%)	1.72(0.16–19.12)	0.653
UAE	13	10(76.9%)	1.03(0.28–3.79)	0.962
Iraqi	17	3(17.6%)	17.31(4.92–60.92)	** < 0.001**
Israeli	14	8(57.1%)	2.63(0.90–7.69)	0.066
Kuwaiti	54	27(50.0%)	3.87(2.20–6.78)	** < 0.001**
Libyan	9	4(44.4%)	4.39(1.17–16.53)	**0.017**
Malaysian	25	13(52.0%)	3.33(1.49 -7.43)	**0.002**
Palestinian	9	9(100.0%)	NC	0.125
Qatari	6	6(100.0%)	NC	0.185
Saudi	95	31(32.6%)	10.41(6.49–16.69)	** < 0.001**
Syrian	12	9(75.0%)	1.15(0.31–4.29)	0.836
Yemeni	2	1(50.0%)	3.45(0.22–55.48)	0.352
Egyptian	1	0(0.0%)	4.46(3.92 -5.07)	0.063
Total	808	626(77.5%)		

Abbreviations: *NC* Could not be Calculated * Pearson chi-square

High school admission grades were the highest for students with Saudi high school (97.5 ± 3.0%) followed by Yemeni (97.4 ± 1.7%), UAE (97.4 ± 3.3%), Jordanian (96.9 ± 2.8%), Malaysian (96.2 ± 0.8%), IG (95.4 ± 2.4%), Palestine (95.3 ± 4.2%), Qatari (95.0 ± 2.7%), Israeli (94.8 ± 3.2%), Syrian (94.6 ± 4.5%), IB (93.9 ± 2.5%), Kuwaiti (93.4 ± 3.7%), SAT (93.3 ± 4.6%), Bahreini (93.2 ± 2.6%), Libyan (92.5 ± 5.6%), Canadian (91.4 ± 0.9%), Egyptian (91.2 ± 0.0%), and Iraqi (90.5 ± 3.5%) high school degrees (Fig. 1). Students admitted with Jordanian high school and IB high school grades were more likely to graduate than the rest of students (*p* < 0.001 and *p* < 0.029 respectively). On the other hand students admitted with Iraqi (*P* < 0.001), Kuwaiti (*P* < 0.001), Libyan (*P* < 0.017), Malaysian (*P* < 0.002), and Saudi(*P* < 0.001) high school were less likely to graduate than the rest of students (Table 4).

The students that graduated with the highest GPA where those that entered the program with the IB (GPA 3.27 ± 0.14; *P* < 0.007), IGCSE (GPA 3.17 ± 0.07; *p* < 0.001), SAT (GPA 3.23 ± 0.18; *P* < 0.105) and Jordanian high school degrees (GPA 3.09 ± 0.02; *P* < 0.001). Also, students coming with Jordanian (JGSC) and IB high school degrees graduated from the medical school with significantly higher GPA than students coming with Saudi, Kuwaiti and Qatari high school degrees (Table 5).

**Table 5:** Association between high school programs and graduating GPA

High school degree	Graduating GPA Mean(SE)^a^	Correlation between graduating GPA and high school admission grades	
		Pearson Correlation	*P* value
JGSC	3.09(0.02)^s,k,q^	0.476	** < 0.001**
IGCSG	3.17(0.07)^s,k,q^	0.479	** < 0.001**
SAT	3.23(0.18)	0.576	0.105
IB	3.27(0.14)^s,k,q^	0.647	**0.007**
Bahrein	2.81(0.08)	0.625	0.098
Canada	2.71(0.22)	1	
UAE	2.97(0.19)	0.451	0.191
Irak	2.76(0.20)	0.86	0.341
Israel	3.01(0.17)	0.579	0.133
Kuweit	2.68(0.08)^j, i, b^	0.469	**0.014**
Lybia	2.80(0.26)	0.381	0.619
Malysia	2.73(0.07)	0.233	0.443
Palestine	3.00(0.20)	0.604	0.085
Qatar	2.44(0.08) ^j, i, b^	-0.059	0.912
Saudi	2.76(0.07) ^j, i, b^	0.225	0.223
Syria	3.09(0.17)	0.4	0.285
Yemen			

^a^ Statistical differences between high school degrees were assessed with Kruskal Wallis test; Abbreviations: s: significantly different from Saudi degrees; K: significantly different from Kuwaiti degrees; Q significantly different from Qatari degrees, JGSC significantly different from Jordanian Secondary general exam; I: significantly different from IGCSE; b: significantly different from IB

The graduating GPA was significantly correlated to the high school scores for students coming with IGCSE, JGSC, IB and Kuwaiti highs school degrees, but not for students coming with other high school degrees (Table 5).

## Discussion

This study showed how admission criteria such as admission pathways and the high school degrees could be strongly associated with student success in a medical school in terms of graduation rates and final GPA. The wide background of the students admitted at The Faculty of Medicine of Jordan University allowed for assessment of how different high school degrees and admission systems could influence student performance in the medical school. Students admitted through more competitive quota such as the open competition and the quota for underprivileged students performed better than students admitted through less competitive quota such as international students and for students admitted through the parallel system. Regarding the high school degree, students admitted with IB, IGCSE, SAT and Jordanian high school had the best performance in the program in terms of GPA and graduation rates. Underneath we discuss our findings in detail.

In our study the dropout rate of the admitted students was relatively high (22.5%) compared with a recent meta-analysis reporting an average dropout rate of 11.1% (2.4%- 26.2%) [19]. However, this rate varied depending on the admission pathway and the high school certificate of the admitted students. Students with Jordanian GCS had a dropout rate of just 6.8% while students with high school degrees from other countries such as Kuwait or Saudi Arabia had higher dropout rates. Similar trends were observed with the graduating GPA. Students coming with IGCSE, Jordanian General Secondary School Certificate (JGSC; a.k.a “Tawjihi”), IB and SAT high school degrees seemed to graduate from the medical school with higher GPA. Moreover, IGCSE, JGSC, IB and Kuwaiti high school grades seemed to be good predictors of student performance in the medical program. Such correlation was not observed for other high school degrees, probably because we had very few students coming with those degrees. One exception was the Saudi high school degree that included as many as 31 students, but it still failed to show a correlation with graduating GPA.

These differences between high school degrees could be due to the way the exams are weighed. For instance, the Jordanian JGSC, the IB, the SAT and the IGCSG, relay exclusively on highly standardized exams performed in accredited sites. On the other hand, other regional programs such as the Kuwait, the Qatari and the Saudi Arabia high school certificates rely heavily on grades provided by the schools attended by the students, which could compromise the objectiveness of the assessments [20, 21]. Therefore, it could be recommended for medical schools to review the requirements of students admitted with certain high school degrees. Perhaps these students would need additional cognitive assessment before their admission into a medical school.

Within the limits of our knowledge, this could be the first study reporting the correlations between a wide range of high school programs and the graduation GPA of medical students. In our study, IB students had the highest graduating GPA, and IB scores showed the strongest correlation with graduating GPA. This observation agrees with another study on students from the university of Florida showing that IB students consistently earned the highest mean GPA compared to students admitted with other high school degrees [22]. In our study SAT students graduated from the medical school with some of the highest GPAs, however the SAT score and the graduation GPA did not correlate well for SAT students. One study on 161 freshman students found that the SAT and ACT could predict freshman GPAs and suggested that both the SAT and ACT are strongly related to IQ and intelligence tests [23]. Another study showed that SAT scores are good predictors for success in the College of Principles of Economics at the University of South Carolina [24].

The admission quota in our study favored international students as well as those admitted through the parallel and the “other” pathways. However, even though these students were probably from a privileged background (they were able to pay much higher tuition fees) they had lower high school grades and were less likely to graduate from the medical school. On the other hand, fewer students were admitted through open admission and the pathway for underprivileged students (i.e. makrumah), even though they had the higher high school grades and they were the most likely to graduate.

A study in Germany compared the performance of 655 medical students according to their admission pathway (university-specific selection quota, pre-university GPA quota, waiting time quota, ex-ante quota and foreign students). The study found that students admitted through the pre-university GPA quota and the university specific selection quota performed better than students in the waiting time and ex-ante quotas while foreign students had the weakest performance [25]. Another study on 1565 medical students enrolled in Hamburg from 2012 to 2015 showed that students admitted through the entrance test or the quota for excellent pre-university educational attainment performed markedly better during the first 3 terms than students admitted through the waiting list quota and the quota for foreign students [16].

Socially responsible medical schools aim to reduce health inequalities by training a workforce able to address the needs of underserved communities; to better serve society, medical schools need to have a population of students and staff that are representative of the society they serve. One key strategy involves recruiting students from underserved and unequally represented communities on the basis that they may be more likely to return to their local communities and better address their health priorities [26]. This is achieved by a fair and equitable representation of underserved communities within the student body. Such strategies may contribute to a diverse medical student body with strong intentions to work with underserved populations [26]. In order to improve the distribution and shortage of physicians in rural areas Japan stablished a quota for students coming from those areas. However, this system has shown problems as many students end up not serving in rural areas after graduation [27].

In middle eastern societies, education, and especially medical education, is a main driver for social mobility. However, privileged access to the medical profession by members of the dominant strata of the society limits social mobility and produces healthcare professionals out of touch with the broader sector of the society they are supposed to serve. For this reason, many medical schools have moved from simple allocation of admission quota for disadvantaged students, towards addressing deeper questions about what kind of excellence is expected from applicants, how to measure it, and how could applicants improve the care for the communities they come from [28]. Equity-based approaches for student recruitment in medical schools needs to move beyond the recruiting students from marginalized backgrounds who happen to resemble the most the dominant segment of the society. This requires a more complex approach that recognizes how disadvantage could be experienced in many different ways, that there is a wide spectrum of access barriers, and that equity can only be achieved when institutions embed critically, socially conscious approaches throughout their practices [28].

Unfortunately, in Jordan University Faculty of Medicine the admission pathways are severely skewed towards the privileged segments of the society that can pay higher fees for access through the parallel admission system, and the international student quota. Students admitted through the open admission system and the makrumah system are the only ones that are eligible for more affordable tuition fees, but they were fewer than 30% of all admitted students.

Besides socioeconomic disparities, in our study we also observed gender disparities in the admission process. We observed that more females were admitted through the more competitive admission pathways such as the national unified system, while more male students joined through the less competitive admission pathways (i.e. parallel and the international pathways). The large number of female students admitted through the competitive pathways is expected because female students achieve overall higher highs school grades in Jordan. Thus, the lower numbers of female students joining through the least competitive pathways could be more related to socioeconomic factors rather than academic factors. These differences could be related to the fact that in the middle east female students are less likely to travel to continue their studies, however, the differences remained significant after adjustment for the high school degree type. This seems to indicate that tuition fees may also be playing a role in the gender differences during the admission process. It could be possible that families were more likely to pay the more expensive tuition fees of the parallel and international admission pathways for their male children than for their female children.

Despite the gender differences in the admission process, male and female students performed in a similar way once admitted to the school. There were no significant differences in the graduating GPA between male and female students with the exception of “Makruma” students, among whom male students performed better. The role of gender on student performance in medical schools seems to be controversial [4, 13, 14, 16]. Some studies report that female students are overrepresented and tend to outperform their male colleagues [4, 14]. In contrast, some reports revealed a better performance by male students [13, 16], and other studies observed a similar performance between genders [14]. Our results seem to indicate that gender differences could be associated to the socioeconomic status of the students.

### Strengths and limitations

This comprehensive study includes students from different demographic and academic backgrounds, coming from 14 different countries, and holding 17 different types of secondary school certificates. In addition, it includes the academic achievements before admission to medical school and upon graduation. However, our study has some limitations. Our sample was obtained from a single institution, the sample size was relatively small, and it did not analyze the socioeconomic status of the students. Moreover, this research was limited to the academic performance in the medical school and did not analyze the long-term professional performance.

## Conclusion

Admission criteria such as type of high school degree and grades as well as admission pathways can predict likelihood to graduate and graduation GPA of medical students. Open competition admission pathways as well as IB, IGCSE and Jordanian high school degrees seem predict better student performance in a Jordanian medical school.

## Supplementary Information

**Additional file 1: Supplementary Table 1.** Characteristics of students accepted in the academic year 2012-2013.

**Additional file 2: Supplementary Table 2.** Characteristics of students accepted in the academic year 2013-2014.

## Data Availability

The datasets used and/or analyzed during the current study are available in the office of the corresponding author on reasonable request.

## Abbreviations

ACT: American College Testing
JGSC: Jordanian General secondary certificate
GPA: Grade point average Degree
IQ: Intelligence Quotient
IB: International baccalaureate
IGCSE: International general secondary certificate of secondary education
I: International track
IRB: Institutional Review Board
M: Makrumah
MD: Medical Degree
NUA: National Unified Admission
O: Others Track
P: Parallel track
SAT: Scholastic Assessment test
UEC: Children of university employees track
UAE: United Arab Emirates
USMLE: United States Medical Licensing Examination
